# A Model for Evaluating the Performance of a Multiple Keywords Spotting System for the Transcription of Historical Handwritten Documents

**DOI:** 10.3390/jimaging6110117

**Published:** 2020-11-03

**Authors:** Angelo Marcelli, Giuseppe De Gregorio, Adolfo Santoro

**Affiliations:** 1 Natural Computation Lab, Dipartimento di Ingegneria dell’Informazione ed Elettrica e Matematica Applicata, University of Salerno, 84084 Fisciano, Italy; amarcelli@unisa.it; 2 Natural Intelligent Technologies, 84084 Penta, Italy; asantoro@nitesrl.com

**Keywords:** historical document processing, keyword spotting, performance evaluation models

## Abstract

This paper proposes a performance model for estimating the user time needed to transcribe small collections of handwritten documents using a keyword spotting system (KWS) that provides a number of possible transcriptions for each word image. The model assumes that only information obtained from a small training set is available, and establishes the constraints on the performance measures to achieve a reduction of the time for transcribing the content with respect to the time required by human experts. The model is complemented with a procedure for computing the parameters of the model and eventually estimating the improvement of the time to achieve a complete and error-free transcription of the documents.

## 1. Introduction

Digital libraries have evolved from a way to store and preserve documents to an integrated platform of information processing and web applications for allowing preservation, creation, and manipulation of information and knowledge. This has required the development of specialized software tools for processing the document digital images in order to extract their textual content in a computer-readable format.

A large amount of this cultural heritage is available in the form of small collections of handwritten documents, typically containing less than 1000 pages written by a few different writers, stored in local museums and churches archives, notary documents, and business contracts and accounts, and access to their content is of paramount importance for depicting the evolution of the cultural, social, political, and economic circumstances of life in a specific region.

Those collections are of particular interest for historians, but, as they became available in their digital form on the website of the above-mentioned institutions and organizations, the general public has also become interested in accessing their content, searching for family ancestors or events whose knowledge was reported orally and vaguely. Therefore, making their content accessible has become a more and more pressing demand, and so librarians and public administrators have turned their attention towards computer-assisted transcription, as it promises to be faster and cheaper than human methods.

In the case of historical handwritten documents’ transcription, the enabling technologies are handwriting recognition and keyword spotting. The goal of handwriting recognition is to correctly classify a word image into a labeled class, or else obtain its transcription. As they have to deal with the huge handwriting variability encountered in different collections produced by many writers, they rely on complex tools, such as hidden Markov models, conditional random fields, and artificial neural networks, and often resort to hybrid approaches by combining different tools [1]. To achieve high accuracy, however, they need huge training sets, usually in the thousands of pages, and have been successfully adopted for large collections, often exploiting crowdsourcing for labelling the training sets in order to make the overall cost acceptable [2].

Keyword spotting (KWS), also known as recognition-free retrieval, has drawn the attention of the research community, in that they circumvent the drawbacks of explicit recognition [3,4]. Keyword spotting was initially proposed in the speech recognition community [5], but later on it was adopted for printed document [6], handwritten music scores [7], and technical drawings as well [8]. It is essentially a matching process between the images of a training set, whose transcription is also known, and the images of the document to be transcribed.

Keyword spotting techniques can be grouped depending on whether the keyword is a string of characters (*Query-by-String*) [9,10,11,12,13,14,15] or a word image (*Query-by-Example*) [16,17,18,19,20,21,22] and whether the region of the document image to label is made by a preliminary segmentation step (*segmentation-based*) [23,24,25,26] or the whole page image (*segmentation-free*) [27,28,29]. For the purpose of historical document transcription, however, the most relevant discrimination is between *lexicon-based* and *lexicon-free* approaches. The former relies on the presence of a predefined keyword list, fixed during the training [9,12,23,30,31,32,33,34], while the latter does not rely on such a list [35,36,37,38,39,40], or can find new keywords, as has been recently proposed [41,42].

Considering that, regardless of the technology adopted for implementing the system, user intervention is mandatory to validate and/or to correct the system output to achieve a complete and error-free transcription of the document content, we are interested in establishing the minimum requirements on the performance of the KWS so as to make using the system advantageous to reduce the user time to achieve the complete transcription of the document. In other words, we would like to answer the following questions: Is the KWS system good enough so that the user time required to validate its output in order to achieve the complete and correct transcription of the document content is smaller than the user time required for the manual transcription? In the affirmative, can we estimate how much is the user time reduced?

In our previous work, we have addressed this problem considering the case of a KWS system that provides as output only the best matching keyword. We have derived the conditions under which the use of the system is profitable and introduced a procedure for estimating both the performance improvements and the accuracy of the estimate with respect to the actual improvement [43]. In this paper, we present a model to deal with the most general case of a KWS whose output for each word image is a ranked list of its possible transcriptions.

Regarding the rest of the paper, in Section 2, we summarize the framework for developing our performance model and then derive the expressions of the model for both lexicon-based and lexicon-free KWS. Then, in Section 3, we show how the model can be used for estimating the user time reduction in both cases. Eventually, we conclude by discussing the paramount importance of the size and composition of the training set and suggest a way to build it so as to perform a quick and cheap preliminary evaluation of the possible advantage of using the system, and outline our future efforts for establishing whether the model can provide the bounds for the actual reduction of the user time.

## 2. The Model

In order to answer the previous question, we assume the following:

- We are using a segmentation-based KWS system. This requires that the collection of documents we want to transcribe has been segmented to extract $n_{DC}$ images, each containing one word;
- The KWS system provides, for each word images, an output list containing a ranked list of k possible transcriptions;
- $n_{TS}$ images of the data collection have been manually transcribed and used as training set (*TS*), so that the number of samples that compose the data set (*DS*) to transcribe for completing the task is $n_{DS}=n_{DC}-n_{TS}$. In the case in which a *Query-by-Example* KWS system is used, the transcriptions are needed so that they can be automatically associated to the images retrieved by the system, while in case of a *Query-by-String* KWS system, the transcriptions are needed to train the system during the supervised learning step they envisage. In the following, we will denote with $T_{TS}$ the time needed to choose the images of *TS* and to enter their transcriptions;
- The query list, i.e., the complete list of keywords to spot, is not available, as is customary in KWS performance evaluation literature, but rather, the only available information is obtained by transcribing the training set. Denoting with *N_DC_* and *N_TS_* the number of keywords, i.e., the number of entries in the vocabulary associated to *DS* and *TS*, respectively, this means that *N_TS_* is known, because of the manual transcription of the samples in the training set, while *N_DC_* is not known.

We can now express the time for manually transcribing the whole data collection as the sum of the time for transcribing the word images of *TS* and the time for transcribing the word images of *DS*:

(1) $$T_{man}=T_{TS}+T_{DS}$$

When using a KWS for transcribing the document content, the time $T_{u}$ spent by the user for achieving the complete transcription can be expressed as follows:

(2) $$T_{u}=T_{TS}+T_{out}+T_{miss}+T_{oov}$$

where $T_{TS}$ is the same as the above; $T_{out}$ is the time to validate or correct the system outputs; $T_{miss}$ is the time for manually transcribing the missed words; and $T_{oov}$ is the time for manually transcribing the word images that are instances of out-of-vocabulary (OOV) keywords, i.e., keywords that are not included in the query list. Thus, the use of the *KWS* is profitable when

(3) $$T_{u}<T_{man}$$

which can be expressed as

(4) $$T_{out}+T_{miss}+T_{oov}<T_{DS}$$

The measures used in literature to evaluate the performance of an information retrieval system that provides as output k alternatives are the recall@k and the precision@k:

(5) $$recall@k=R^{k}=\frac{\#Retrieved\;Relevant\;Images\;at\;k}{\#Relevant\;Images}$$

(6) $$precision@k=P^{k}\;\frac{\#Retrieved\;Relevant\;Images\;at\;k}{\#Retrieved\;Images}$$

Let us now denote the following:

- $r_{i}^{k}$ and $p_{i}^{k}$ are the recall@k and precision@k of the KWS for the *i-th* keyword computed on *DS*;
- *n_i_^DS^* is the number of word images of the *i-th* keyword in *DS*;
- $n_{i}^{c}$ is the number of correct samples, i.e., the number of word images of *DS* that are instances of the *i-th* keyword and whose output list includes that keyword;
- $n_{i}^{w}$ is the number of wrong samples, i.e., the number of word images of *DS* that are not instances of the *i-th* keyword, but whose output list includes that keyword;
- $n_{i}^{m}$ is the number of missed samples, i.e., the number of word images in *DS* that are instances of the *i-th* keyword, but whose output list does not include that keyword;
- $n_{i}^{OOV}$ is the number of *out-of-vocabulary* samples, i.e., the number of word images of *DS* that are instances of the *N_DS_* = *N_DC_* − *N_TS_* unknown entries of the keywords list of the data set.

We can estimate for each keyword how the system outputs will be distributed among correct, wrong, and missed words as function of its performance:

(7) $$n_{i}^{c}=r_{i}^{k}\times n_{i}^{DS}$$

(8) $$n_{i}^{w}=\left(\frac{1}{p_{i}^{k}}-1\right)\times r_{i}^{k}\times n_{i}^{DS}$$

(9) $$n_{i}^{m}=\left(1-r_{i}^{k}\right)\times n_{i}^{DS}$$

Denoting with $t_{i}^{v}$ the time required to validate a correct sample of the *i-th* keyword, with $t_{i}^{w}$ the time required to provide the correct transcription for a wrong sample of the *i-th* keyword, with $t_{i}^{m}$ the time required to provide the transcription of a missed sample of the *i-th* keyword, and with $t_{i}^{M}$ the time required to manually transcribe a word image, we can write the expression for each of the four terms in Equation (4) as follows:

(10) $$T_{out}=\overset{N_{TS}}{\underset{i=1}{\sum }}\left[\left(t_{i}^{v}\times n_{i}^{c}\right)+\left(t_{i}^{w}\times n_{i}^{w}\right)\right]=\overset{N_{TS}}{\underset{i=1}{\sum }}\left[\left(t_{i}^{v}\times r_{i}^{k}\right)+\left(t_{i}^{w}\times \left(\frac{1}{p_{i}^{k}}-1\right)\times r_{i}^{k}\right)\times n_{i}^{DS}\right]$$

(11) $$T_{miss}=\overset{N_{TS}}{\underset{i=1}{\sum }}\left(t_{i}^{m}\times n_{i}^{m}\right)=\overset{N_{TS}}{\underset{i=1}{\sum }}\left(t_{i}^{m}\times \left(1-r_{i}^{k}\right)\times n_{i}^{DS}\right)$$

(12) $$T_{oov}=\overset{N_{DS}}{\underset{i=N_{TS+1}}{\sum }}t_{i}^{M}\times n_{i}^{DS}$$

(13) $$T_{DS}=\overset{N_{TS}}{\underset{i=1}{\sum }}t_{i}^{M}\times n_{i}^{DS}+\overset{N_{DS}}{\underset{i=N_{TS+1}}{\sum }}t_{i}^{M}\times n_{i}^{DS}$$

Thus, $T_{out}$ and $T_{miss}$ depend on the system performance as well as on the type of output it provides, as will be shown in the following subsections. On the contrary, $T_{oov}$ depends only on the size and composition of the training set; for a given number of training samples, the larger *N_TS_*, the smaller the difference *N_DS_* − *N_TS_*, and thus the smaller $T_{oov}$.

Equations (10)–(12) point out that the effects of the system performance on the user time, however, are modulated by the times to validate, correct, and transcribe the correct, wrong, and missed samples that depend on the user interface of the system. Thus, given the system performance in terms of $\;r_{i}^{k}$ and $\;p_{i}^{k}$ and the time $t_{i}^{M}$, Equation (4) allows to establish the maximum values of $t_{i}^{v}$, $t_{i}^{w}$, and $t_{i}^{m}$ that satisfy the condition for a profitable use of the system to achieve the complete transcription of the data set. Conversely, given the characteristics of the user interface in terms of $t_{i}^{v}$, $t_{i}^{w}$, and $t_{i}^{m}$, the equation allows to calculate the minimum values of $r_{i}^{k}$ and $p_{i}^{k}$ that must be exhibited by the KWS system to be profitable in assisting the transcription. In the following subsections, we will derive such conditions for lexicon-based and lexicon-free KWS systems.

### 2.1. Lexicon-Based Systems

In this case, as the KWS system is not able to find *OOV* words, the use of the system is profitable when

(14) $$T_{out}+T_{miss}<T_{DS}-T_{oov}$$

which can be written in terms of the KWS system performance as

(15) $$\overset{N_{TS}}{\underset{i=1}{\sum }}\left[\left(t_{i}^{v}\times r_{i}^{k}+t_{i}^{w}\times r_{i}^{k}\times \left(\frac{1}{p_{i}^{k}}-1\right)+t_{i}^{m}\times \left(1-r_{i}^{k}\right)\right)\times n_{i}^{DS}\right]<\overset{N_{TS}}{\underset{i=1}{\sum }}t_{i}^{M}\times n_{i}^{DS}$$

In the case of a perfect KWS system, i.e., a system for whom $r_{i}^{k}=p_{i}^{k}=1\;\forall i,$ the inequality above becomes

(16) $$\overset{N_{TS}}{\underset{i=1}{\sum }}t_{i}^{v}\times n_{i}^{DS}\;<\;\overset{N_{TS}}{\underset{i=1}{\sum }}t_{i}^{M}\times n_{i}^{DS}$$

which certainly holds if $t_{i}^{v}<t_{i}^{M}\;\forall i$. In the case of a real system, both $\;r_{i}^{k}$ and $\;p_{i}^{k}$ are smaller than 1 and the first and last term of the sum on the left side of the inequality (15) show an opposite trend; that is, the former becomes larger, while the latter becomes smaller as $\;r_{i}^{k}$ increases. The second term has a more complex behavior, but because $\frac{1}{\;p_{i}^{k}}>1$, and considering that, in any information retrieval system, recall and precision are such that if $\;p_{i}^{k}$ increases $\;r_{i}^{k}$ does not (and usually decreases), it becomes smaller as $\;p_{i}^{k}$ increases.

### 2.2. Lexicon-Free Systems

Let us now consider the case when the KWS system is able to spot word images whose transcription is not in the query list. To estimate to which extent the system is able to spot *OOV* words, we assume that a test set (*TSS*) containing $n_{TSS}\;\approx \;n_{TS}$ samples of the data collection is provided to the KWS system trained on *TS* as the data set to be transcribed.

We can divide the *OOV* found by the KWS system in *TTS* into two parts. These include the correct *OOV*, composed of the *OOV* words that have an empty output list, and the wrong *OOV*, made up of the *OOV* words that have a non-empty output list. Under the same assumptions of the previous subsection, and denoting with $n_{i}^{OOV^{C}}$ and $n_{i}^{OOV^{w}}$, respectively, the number of correct *OOV* and wrong *OOV* word images that are instances of the *N_OOV_* keywords, we can estimate $T_{oov}$ as follows:

(17) $$T_{oov}=\;\overset{N_{OOV}}{\underset{i=N_{TS+1}}{\sum }}\left(t_{i}^{M}\times \;n_{i}^{OOV^{c}}+\;t_{i}^{Mw}\times n_{i}^{OOV^{w}}\right)$$

It is worth noting that $t_{i}^{Mw}>t_{i}^{M}$ because, in the case of wrong *OOV*, the user needs to read the output list to search for the transcription, and only afterwards will start transcribing the word. On the contrary, the time for transcribing the correct *OOV* is just the time for its transcription, because the output list is empty.

At this point, the user after interacting with the system has spent the time:

(18) $$T_{u}=T_{out}+T_{miss}+T_{oov}$$

where the times appearing on the right side are estimated using the Equations (15) and (17), respectively, and have a query list of $N_{TTS}=N_{TS}+N_{OOV}$ keywords. Thus, to achieve the transcription of the remaining samples of the data set, the user will spend the time $T_{out}^{\prime }$ for processing the output of the system and the time $T_{miss}^{\prime }$ for transcribing the missed words when spotting the $N_{TTS}$ keywords, plus the time $T_{oov}^{\prime }$ for transcribing the *OOV* word, i.e., the word images that are instances of the $N_{DS}-N_{TTS}$ keywords. We can express these times as follows:

(19) $$T_{out}^{\prime }=\;\overset{N_{TTS}}{\underset{i=N_{TS}+1}{\sum }}\left[\;\left(t_{i}^{v}+\;t_{i}^{w}\times \left(\frac{1}{\;p_{i}^{k}}-1\right)\;\right)\right]\times \;r_{i}^{ki}\;\times \;n_{i}^{DS}$$

(20) $$\;T_{miss}^{\prime }=\;\overset{N_{TTS}}{\underset{i=N_{TS}+1}{\sum }}\left(t_{i}^{m}\times \;\left(1-\;r_{i}^{k}\right)\times n_{i}^{DS}\right)\;$$

(21) $$\;T_{oov}^{\prime }=\;\overset{N_{DS}}{\underset{i=N_{TTS+1}}{\sum }}\left(t_{i}^{M}\times \;n_{i}^{OOV^{c}}+\;t_{i}^{Mw}\times \;n_{i}^{OOV^{w}}\right)\;$$

and thus

(22) $$T_{u}^{\prime }=\;T_{out}^{\prime }+T_{miss}^{\prime }+T_{oov}^{\prime }$$

To estimate $T_{oov}^{\prime }$, we need the value of $N_{DS}$ as well as those of $n_{i}^{OOV^{c}}$ and $n_{i}^{OOV^{w}}$. We can estimate them by assuming that the coverage of the query list computed on *TTS* with respect to the actual one, i.e., the ratio $\frac{N_{TTS}}{N_{DS}}$ is the same as the coverage of the query list computed on *TS* with respect to the one of *TTS*, i.e., the $\frac{N_{TS}}{N_{TTS}}$ ratio, and thus $N_{DS}=\frac{N_{TTS}^{2}}{N_{TS}}$. Similarly, we estimate $n_{i}^{OOV^{c}}$ and $n_{i}^{OOV^{w}}$ by assuming that the distribution of the *OOV* words between correct and wrong in *DS* is the same as it was in *TTS*.

Under these assumptions, we can split the time $T_{DS}$ for the manual transcription of *DS* into the time for transcribing the word images that are instances of the *N_TS_* keywords obtained from the manual transcription of *TS*, the time for transcribing the word images that are instances of the *N_TTS_* keywords obtained from the data in *TTS*, and the time for the transcription of the remaining *OOV* words:

(23) $$T_{DS}=\overset{N_{TS}}{\underset{i=1}{\sum }}t_{i}^{M}\times \;n_{i}^{DS}+\;\overset{N_{TTS}}{\underset{i=N_{TS}+1}{\sum }}t_{i}^{M}\times \;n_{i}^{DS}+\overset{N_{DS}}{\underset{i=N_{TTS+1}}{\sum }}t_{i}^{M}\times \;n_{i}^{DS}$$

We can now establish the condition for the profitable use of a lexicon-free KWS as follows:

(24) $$T_{u}+T_{u}^{\prime }<\;T_{DS}$$

This expression shows that using the KWS system may be profitable with respect to the manual transcription if inequality (24) holds for the word images that are instances of the keywords of the query list to such an extent to compensate for the extra time due to the transcription of the wrong *OOV* words spotted by the system, i.e., when the following occurs:

(25) $$\left(\overset{N_{TTS}}{\underset{i=1}{\sum }}t_{i}^{M}\times n_{i}^{DS}-\overset{N_{TSS}}{\underset{i=1}{\sum }}\left[\;\;\right]\times \;n_{i}^{DS}\right)\;<\;T_{oov}+\;T_{oov}^{\prime }$$

where the expression between the square bracket is the same as in (15).

## 3. The Model at Work

To show how to use the model in practice, let us define the gain *G* achievable while using the keyword spotting system as in [43]:

(26) $$G=1-\frac{T_{user}}{T_{man}}$$

where $T_{user}$ = $T_{u}$ in the case of a lexicon-based *KWS* or $T_{user}\;=\;T_{u}+T_{u}^{\prime }$ in the case of a lexicon-free one, and $T_{man}$ is defined as in the previous section.

The parameters of the models described in the previous section can be computed or estimated by performing the following steps:

### 3.1. Transcription of the Training Data

This step requires to transcribe manually the word images of the training set and recording the time to achieve a complete and correct transcription. After the training set is manually transcribed and the time spent by the user is recorded, we know the values of $n_{i}^{TS}$, *N_TS_*, and $t_{i}^{M}$ for each keyword.

### 3.2. Training of the System and Feasibility Check

After training of the system, it is possible to obtain for each keyword the values $r_{i}^{k}$ and $p_{i}^{k}$ computed on *TS* and to check whether or not condition (15) holds. If this is not the case, and considering that the values $r_{i}^{k}$ and $p_{i}^{k}$ on *DS* are very likely to be smaller than those computed on *TS*, the performance of the KWS system may not good enough for profitable use of the assisted transcription instead of the manual one. At this point, it is possible to consider enlarging the training set or, if possible, to reconfigure the KWS system with a larger value of *k* and to repeat the check. The first approach requires more user time, while the second depends on the architecture of the KWS.

### 3.3. Keyword Spotting on the Test Set

Once the KWS system has been trained and has passed the feasibility check, it is used to spot the words of *TTS*. After validating the system outputs, we achieve the transcription of the test set; obtain the values of $t_{i}^{v}$, $t_{i}^{w},\;\mathrm{and}$ $t_{i}^{m};$ and can compute the values of $r_{i}^{k}$ and $p_{i}^{k}$. In the case of a lexicon-free system, we can also obtain the values for $N_{TTS}$, $n_{i}^{OOV^{c}}$, and $n_{i}^{OOV^{w}}$ and compute the values of $t_{i}^{M},t_{i}^{Mw},\;r_{i}^{k}$, and $p_{i}^{k}$ for the $N_{TTS}$ keywords.

#### 3.3.1. Estimating the User Time: Lexicon-Based System

The application of the model described in the previous section requires the values of its parameters as well as the values of $n_{i}^{DS}$ obtained from the data set, which are unknown. Considering that *TS*, *TTS*, and *DS* have been extracted from the data collection we want to transcribe, it is reasonable to assume the following:

1. the distribution of the values of $r_{i}^{k}$ and $p_{i}^{k}$ computed on *TTS* and *DS* is similar;
2. the distribution of the length of the keywords is similar on each set, and because $t_{i}^{M}$ depends mostly on the number of characters rather than on the actual character of the keyword, it is independent of the actual keyword;
3. the values of the model parameters are normally distributed;
4. all the samples of the data set are instances of the keywords obtained from the training set, i.e., that $N_{TS}=\;N_{DS}$.

According to these assumptions, we use for e the mean value computed on *TS*, while for $t_{i}^{v},t_{i}^{w},\;t_{i}^{m},r_{i}^{k},$ and $p_{i}^{k}$, we use the mean values computed on *TTS*, so that the time for processing the system outputs can be written as follows:

(27) $$\left(t^{v}\times \;r^{k}+t^{w}\times \;r^{k}\times \left(\frac{1}{p^{k}}-1\right)\;+t^{m}\times \;\left(1-r^{k}\right)\right)\times \;n^{DS}$$

where all the parameters assume the respective mean values, and use this equation for estimating $T_{u}$.

#### 3.3.2. Estimating the User Time: Lexicon-Free System

In this case, we follow the same line of thought as before for estimating $T_{out}^{\prime }$ and $T_{miss}^{\prime }$ on *TTS*, while the value of $N_{DS}$ as well as those of $n_{i}^{OOV^{c}}$ and $n_{i}^{OOV^{w}}$ can be estimated as in Section 2.2. Under these assumptions, we can estimate $T_{out}^{\prime }$, $T_{miss}^{\prime }$ and $T_{oov}^{\prime }$ as follows:

(28) $$T_{out}^{\prime }=\;\left[\;\left(t^{v}+\;t^{w}\times \left(\frac{1}{p^{k}}-1\right)\;\right)\right]\times \;r^{k}\;\times \;n^{DS}$$

(29) $$\;T_{miss}^{\prime }=\;\left(t^{m}\times \;\left(1-\;r^{k}\right)\times n^{DS}\right)\;$$

(30) $$\;T_{oov}^{\prime }=\;t^{M}\times \;n^{oov^{c}}+\;t^{Mw}\times \;n^{oov^{w}}\;$$

where the values of the parameters are as in the previous case and use them to compute $T_{u}^{\prime }.$

### 3.4. Computing the Gain

Under the same assumptions as before, we can estimate $T_{man}$ as follows:

(31) $$T_{man}=t^{M}\times \left(n_{TS}+\;n_{DS}\right)$$

and eventually derive the estimated value of G, which represents the reduction of the user time achieved using the KWS system with respect to the manual transcription.

## 4. Model Validation

To evaluate if and to which extent the assumptions we made in Section 3.4 allow to obtain a reliable estimate of the actual value of G, we have performed a set of experiments for comparing the estimated value of G with the actual one.

The experiments involved three experts who transcribed the pages of *DS*. The pages were manually transcribed by alternating transcription sessions of 20 min with resting sessions of 10 min, as is customary for avoiding fatigue effects. The transcription sessions were carried out by two experts and, during the resting session, another expert checked for inconsistency between the two transcriptions, so to achieve an error-free transcription. The experts were paleographers with more than 10 years of experience and with basic skills in computer technology, mostly word processing, spell-checking, and annotation tools. Before the transcription, all the experts were trained on the use of the Graphical User interface (GUI) of the tool. There were three training sessions. In the first session, which lasted 60 min, they were introduced to the main features of the GUI for both transcription and validation, while during the two remaining sessions, they were allowed to practice the GUI for transcription and validation, respectively, until they felt comfortable with it. It took less than 30 min for the experts to become familiar with the transcription mode of operation, while it took a little longer, namely minutes, to master the GUI for validation.

The experiments were performed on 50 pages of the Bentham dataset [44], which is a publicly available dataset largely used for assessing the performance of KWS system in international competitions [45,46]. We used 5 pages of the data collection as *TS*, 5 pages as *TTS*, and the remaining 40 pages as *DS*. Table 1 reports the composition of each set.

During each session, we used the user interface to record the expert activity and to eventually compute the value of $t_{i}^{M}$. From the recorded data, we computed the mean μ and the standard deviation σ of these values across the entire data set, obtaining μ=5.81 s and σ=1.237 ms for the first expert, and μ=5.65 s and σ=1.251 ms for the second one. The average value μ=5.73 was then selected as the actual value to be used for estimating the user time. On the 10 pages, there were only 12 words for which the two experts provided different transcriptions. Having assessed the performance of the two experts, each one transcribed half of the 40 pages of *DS* and the sum of the time they spent for transcribing the pages of *DS* and the shortest time for transcribing *TS* and *TTS* was assumed as the time $T_{man}$ for the manual transcription of *DS*. Their values are reported in Table 2.

### 4.1. The Validation Tool

**Figure 1:**
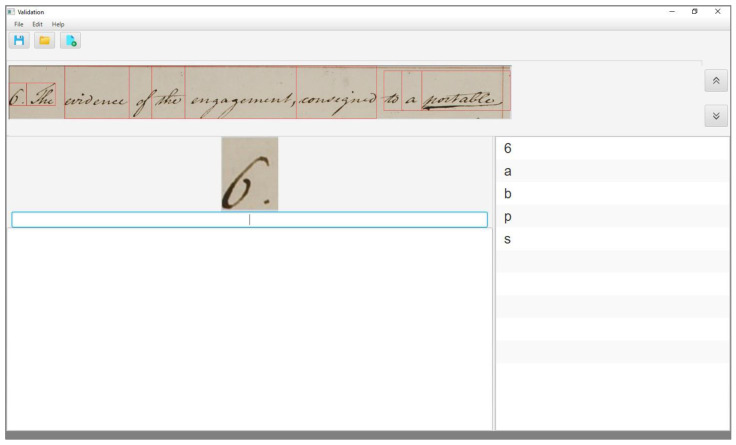
The user interface of the validation tool.

To assess the performance of the KWS system in assisting the transcription, we have designed a validation tool to process the system output. (The validation tool can be obtained free of charge by contacting the corresponding author). As we already mentioned, the values of the times $t_{i}^{v},$ $t_{i}^{w},$ $t_{i}^{m},$ and $t_{i}^{Mw}$ depend on the user interface of the validation tool. In our case, the user interface appears as in Figure 1, once a page of the collection has been opened for validation.

The upper part of the interface shows the current text line of the document, with each word being enclosed within its bounding box, as provided by the segmentation step. In the centre of the interface, the main box shows the current word, i.e., that is being validated and immediately below the box for its manual transcription. The rightmost box shows the output list, and finally, the lowest part of the interface contains a text area to show the transcription of the whole page, line by line, and is updated as the transcription proceeds.

**Figure 2:**
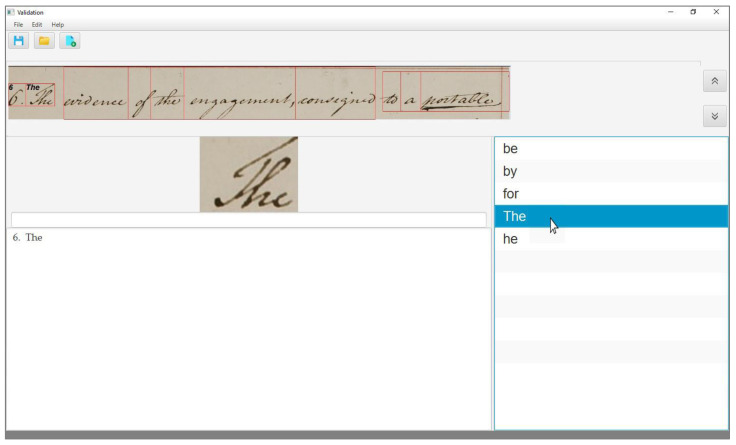
The user interface when transcribing a correct sample.

In the case of a correct sample, the output list contains the correct interpretation. Thus, the user searches for the correct transcription and, once identified, validates the word by clicking on the correct transcription. The interface then shows the transcription on the corresponding word image in the text line as well as in the lowermost box, as shown in Figure 2, and moves to the next word to transcribe.

**Figure 3:**
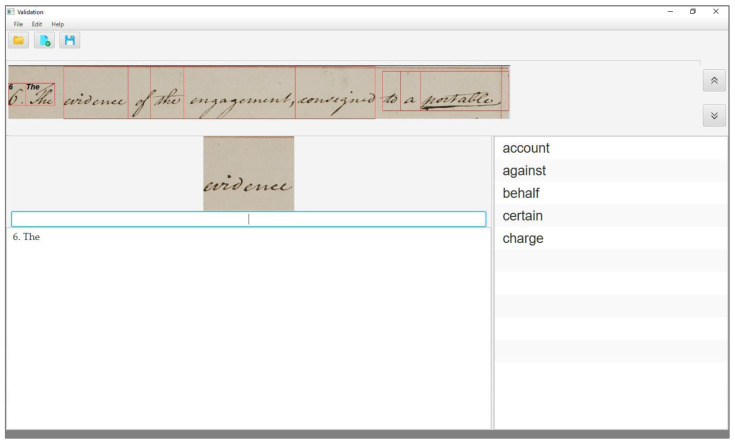
The user interface when transcribing a wrong sample.

**Figure 4:**
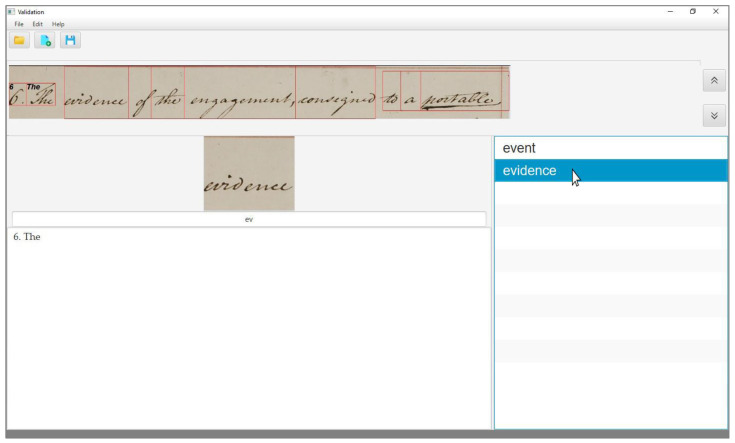
The user interface after a few characters of the word to transcribe have been entered. The output list is updated and, once the correct transcription appears in the box, the user validates the correct transcription by just a click.

In the case of a wrong sample, the correct transcription is not present in the output list, as shown in Figure 3, but the word is an instance of a keyword included in the query list. In this case, the user needs to enter its transcription. To speed up this activity, the interface offers an auto-completion mode; that is, as the first characters are typed by the user, the system updates the output list by showing all the entries of the query list that match with the characters typed so far. Once the correct transcription appears on the interface, as shown in Figure 4, the user can validate it by clicking on it, as in the previous case.

In the case of a missed word, its output list is empty, and thus the user needs to type its transcription manually. As in the previous case, as the user starts typing the characters of the word transcription, the auto-completion mode shows in the output list all the entries of the query list that match the string of characters the user has typed so far, and once the correct one appears, the user can proceed by just clicking on it.

**Figure 5:**
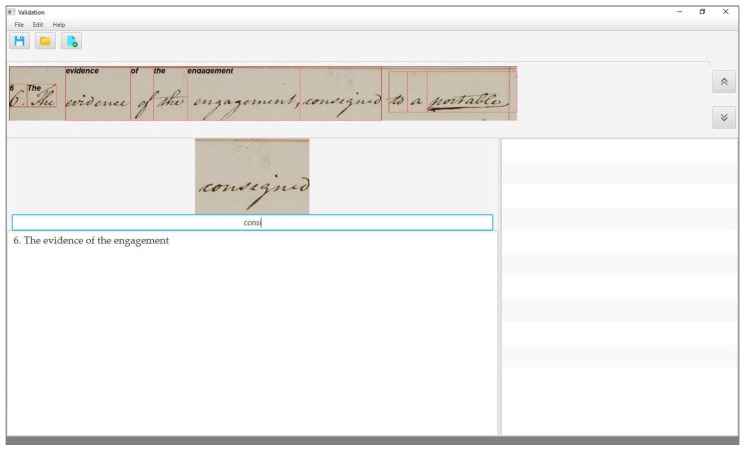
The word “consigned” is a correct *out-of-vocabulary (OOV)* word: the output list is empty and the user enters the entire transcription.

Finally, if the current word is an *OOV* word, the system can either show an empty or a non-empty output list, depending on whether the *OOV* is a correct or a wrong one. In both cases, the user must type in the entire transcription, but in the case of a wrong *OOV*, he will first scan the output list, searching for the transcription, and only afterwards will start to transcribe, as shown in Figure 5.

During the validation sessions, the tool logs all the user action and the time the user spent on each, so that it is possible to compute the number of correct, wrong, missed, *OOV* correct, and *OOV* wrong words as well as the times for achieving their transcriptions.

### 4.2. Experimental Results

In the experimental work, we used our KWS system, whose architecture and mode of operation can be summarized as follows. Basically, it builds on two main blocks: the reference set (*RS*) and the knowledge base (*KB*). *RS* is built by processing the word image of *TS* in such a way to recover the trajectory [47], decomposing it in elementary parts, called strokes [48], and eventually labelling each stroke with the character to which it belongs [49]. Thus, each word is represented by a string of as many characters as the number of strokes extracted from the ink trace. Then, each word of *DS* is decomposed in strokes as before, but the labelling of the stroke is obtained by matching each word of *DS* with all the words of the *RS*: whenever a sequence of strokes whose shape is similar is found, the labels of those strokes found in the words of *RS* are copied to the matching stroke of the word of *DS* [50]. As a result, each word of *DS* is associated with a graph with as many nodes as its number of strokes, and each node is labeled with a character if the corresponding stroke has matched one of the strokes of *RS*. When a query is searched for, the KWS system searches within the graph of each word of *DS* for a path whose nodes correspond to the characters of the query [51]. If such a path is found, the word is returned in response to the query. Because of the multiple labelling of the strokes, it can happen that the same word image is returned in response to different queries, and also that a word that is not an instance of any keywords is spotted, thus allowing the system to spot *OOV* words.

A preliminary experiment was aimed at evaluating if and to what extent the length of the output list was affecting the time for validating the system outputs. The validation tool was configured as to provide the top 5, 10, and 15 top interpretations for each word image. For each configuration, a different expert performed the assisted transcription of a batch of five pages of *DS*. The total time for completing the task was 2996.062 s, 2835.130 s, and 3314.240 s for *k* = 5, 10, and 15, respectively. These results show that, as the length of the output increases, the time for searching the correct transcription in the list counterbalances the improvement of the recall when the output list contains many interpretations. This is in accordance with the observation that human beings can search at a glance within a list of approximately five elements, but when the list becomes longer, the searching time grows almost linearly with the number of elements in the list. These results suggest that, for our user interface, the value 10 represents the best compromise, and thus we have used it in the remaining experiments.

In the second experiment, we performed the keyword spotting on *TTS* and, by recording the user activities, we computed the mean and the standard deviation of the model parameters, as reported in Table 3.

As our KWS is a lexicon-free one, to simulate a lexicon-based system, we disabled the interface to show the output list when the words were *OOV* words (we know that because we have manually transcribed *DS*) and do not update the query list. On the contrary, we enable the displaying of the output list in the case of lexicon-free and add the unique words obtained by the transcription of the *OOV* to the query list. So, by using the values in Table 3, we computed the summation on the left side of the inequality (15), where *n_DS_* was replaced by *n_TT_*_S_. Then, by adding *T_TS_* and *T_OOV_* (using *t^M^* and *t^Mw^* for the lexicon-based and lexicon-free case, respectively), we compute *T_user_* and eventually *G*. We then computed *G* using the actual user time recorded by the tool to complete the task, and then adding *T_TS_*, we computed *T_user_* and the gain *G*. Table 4 reports the value of *T_user_* and *G* estimated using our model and the actual one for both the lexicon-based and the lexicon-free configuration of the *KWS* system.

As shown in Table 4, the model provides a reliable estimate of *G* in both cases, but the one in the case of a lexicon-free system exhibits the largest difference between the estimated and the actual value of *G*.

In the last experiment, the expert who transcribed the first 20 pages of *DS* performed the validation of the system output on the remaining 20 pages, while the second expert who transcribed the last 20 pages validated the system output on the first 20. This procedure was adopted to avoid the memory effect that could have altered the time they spent if they had performed the validation on the same pages they had already transcribed. Table 5 reports the results of the experiment. It shows that, as in the case of *TTS*, the estimated values are an upper bound for the actual ones, but it also shows that the difference between the two remains almost the same, thus confirming that, from the estimates provided by the model on *TTS*, it is possible to draw a reliable estimate of the actual value of G on the entire data collection.

## 5. Conclusions

We have addressed the problem of estimating the reduction of the user time for achieving a complete and correct transcription of small collections of historical documents when a *KWS* system capable of providing multiple possible transcriptions for each word image of the collection is used, in comparison with the user time required by the manual transcription.

The model shows that the user time reduction depends on both the performance of the *KWS* system and the user interface of the validation tool. In particular, it shows that, for a given precision and recall, the actual reduction of the user time depends on the time to process the different types of output (correct, wrong, missed, and *OOV*) with respect to the time for the manual transcription of the corresponding word, in such a way that, the lower the ratio between the time for processing the output and the time for its manual transcription, the higher the reduction of the user time. Conversely, given a user interface to be used for validation, the *KWS* system must exhibit a minimum level of performance to be advantageous in assisting the manual transcription. This interplay between the performance of *KWS* system and the time-efficiency of the user interface should be carefully addressed when designing a system implementing the human-in-the-loop approach to historical document processing.

In the case of a lexicon-based system, the model shows that the benefits due to the performance of the *KWS* are restricted to the word images of the data set that are instances of the keywords of the lexicon; that is, the larger the number of the keywords to spot, the larger the potential benefit. This suggests adopting a multi-step procedure to build a training set containing as many keywords as possible and to divide the data set in batches; in the first step, one batch is used for training the *KWS*, and in each of the following steps, one of the remaining batches is processed. Then, its outputs are validated, and when the user in response to a wrong output enters new keywords, they are added to the keywords list and the next batch is processed. The model also shows that the advantages of using the *KWS* system become larger as the precision increases—even though this may negatively affect the recall—because correcting a wrong word takes longer than transcribing a missed one. Thus, improving the recall at the expense of the precision should be avoided. We have implemented such an approach and are currently performing experiments to evaluate its benefits on the performance of the *KWS* system, as well as on the user reduction time.

In the case of lexicon-free systems, the model shows that, the larger the number of wrong *OOV* spotted by the system, the larger the disadvantage of using the *KWS* system in comparison with both lexicon-based *KWS* and manual transcription, but also that these advantages may be mitigated by the updating the query list. While the mitigation mostly depends on the distribution of the samples of the keyword in the test set and the data set, we speculate that, the higher the precision of the *KWS* system, the lower the number of wrong *OOV* spotted, ensuring the profitability of lexicon-free *KWS* with respect to both lexicon-based and manual transcription. Based on this observation, we are currently working to evaluate to what extent this conjecture is valid, by using different *KWS* systems available in the literature.

At last, but not least, we consider that the values of the model parameters as described in Section 3 are derived from the parameter values obtained from the training and the test sets, under the “reasonable assumption” that, with all the sets being extracted from the data collection and processed by the same system, they exhibit similar statistical behavior. The experimental results have shown that using the mean values obtained on the training and test sets leads to an estimate of *G* that is an upper bound for the actual one. We are currently working on performing similar experiments on different data sets to verify if these results are confirmed and to evaluate how the difference between the estimated values of *G* and the actual one varies depending on the data.

## Figures and Tables

**Table 1:** The composition of the dataset used in the experimental work. *DC*, data collection; *TS*, training set; *TTS*, test set; *DS*, data set.

*n_DC_*	*n_TS_*	*N_TS_*	*n_TTS_*	*N_TTS_*	*n_DS_*
10,733	1089	354	942	391	8702

**Table 2:** Times to manually transcribe the training set, the test set, the dataset, and the whole collection. The times are in milliseconds.

*T_TS_*	*T_TTS_*	*T_DS_*	*T_man_*
6240	5472	52,459	61,534

**Table 3:** The mean and the standard deviation of the model parameters estimated on *TTS*. The times are in milliseconds. *OOV*, *out-of-vocabulary*.

*t^v^*		*t^w^*		*t^m^*		*t^Mw^*		*r^k^*	*p^k^*	*n^oovc^*	*n^oovw^*
μ	σ	μ	σ	Μ	σ	μ	σ				
1024	359	3152	1045	2543	682	5903	2611	0.65	0.71	39	247

**Table 4:** Comparison between the values provided by our model and the actual ones on *TTS*. Times are expressed in the format mm:s.

Values	Lexicon-Based		Lexicon-Free	
**On *TTS***	*T_user_*	*G (%)*	*T_user_*	*G (%)*
*estimated*	62:12	14.86	51:21	20.41
*actual*	64:48	13.52	61:30	15.62

**Table 5:** Comparison between the values provided by our model and the actual ones on *DS*. Times are expressed in the format hh:mm:ss.

Values	Lexicon-Based		Lexicon-Free	
**On DS**	*T_user_*	*G (%)*	*T_user_*	*G (%)*
*estimated*	11:27:37	13.91	10:30:30	19.25
*actual*	11:31:02	12.30	11:10:14	15.23
